# NRAGE Confers Radiation Resistance in 2D and 3D Cell Culture and Poor Outcome in Patients With Esophageal Squamous Cell Carcinoma

**DOI:** 10.3389/fonc.2022.831506

**Published:** 2022-04-01

**Authors:** Huandi Zhou, Guohui Wang, Zhiqing Xiao, Yu Yang, Zhesen Tian, Chen Gao, Xuetao Han, Wei Sun, Liubing Hou, Junling Liu, Xiaoying Xue

**Affiliations:** ^1^ Department of Radiotherapy, Second Hospital of Hebei Medical University, Shijiazhuang, China; ^2^ Department of Central Laboratory, Second Hospital of Hebei Medical University, Shijiazhuang, China

**Keywords:** esophageal squamous cell carcinoma, radioresistance, NRAGE, 3D bio-printing, Wnt/β-catenin

## Abstract

**Objective:**

The purpose of the study is to explore the mechanism of NRAGE enhancing radioresistance of esophageal squamous cell carcinoma (ESCC) in 2D and 3D levels.

**Methods:**

Stably NRAGE-overexpressed ESCC cells and 3D-printing models for ESCC cells were established. Then, cellular malignancy indexes, such as cell morphology, proliferation, radioresistance, motility, apoptosis, cell cycle, and proteins of the Wnt/β-catenin pathway, were compared between radioresistant and its parental cells in 2D and 3D levels. Additionally, 44 paraffin ESCC specimens with radical radiotherapy were selected to examine NRAGE and β-catenin protein expression and analyze the clinical correlation.

**Results:**

Experiments in 2D culture showed that morphology of the Eca109/NRAGE cells was more irregular, elongated spindle-shaped and disappeared polarity. It obtained faster growth ability, stronger resistance to irradiation, enhanced motility, reduced apoptosis ratio and cell cycle rearrangement. Moreover, Western blot results showed β-catenin, p-Gsk-3β and CyclinD1 expressions were induced, while p-β-catenin and Gsk-3β expressions decreased in Eca109/NRAGE cells. Experiments in the 3D-printing model showed Eca109/NRAGE cell-laden 3D scaffolds had the advantage on growth and spheroiding according to the brightfield observation, scanning electron microscopy and Ki-67 IHC staining, and higher expression at the β-catenin protein. Clinical analysis showed that NRAGE expression was higher in tumor tissues than in control tissues of ESCC patients from the Public DataBase. Compared with radiotherapy effective group, both NRAGE total and nuclear and β-catenin nuclear expressions were significantly upregulated from ESCC specimens in invalid group. Further analysis showed a positive and linear correlation between NRAGE nuclear and β-catenin nuclear expressions. Additionally, results from univariate and multivariate analyses revealed NRAGE nuclear expression could serve as a risk factor for ESCC patients receiving radical radiotherapy.

**Conclusion:**

ESCC cells with NRAGE nuclear accumulation demonstrated greater radioresistance, which may be related to the activation of the Wnt/β-catenin signaling pathway. It indicated that NRAGE nuclear expression was a potential biomarker for monitoring radiotherapeutic response.

## Introduction

Esophageal cancer (EC), arising from esophageal epithelial cells, is an epidemic malignancy with conspicuous geographic distribution worldwide. It is fairly well known that China is one of the regions with a high incidence rate of esophageal squamous cell carcinoma (ESCC), which has an enormous burden and is a major histological subtype accounting for 95% of ECs in China (1–4). Statistically, the 5-year overall survival (OS) rate for ESCC is approximately 9–27.1%. Even worse, patients will have poorer prognosis if diagnosed with locally advanced ESCC (5, 6). Radiotherapy (RT) is one of the main treatment methods for ESCC, especially for inoperable and locally advanced ESCC, on which RT plays a crucial role (7). Although the prognosis of patients with ESCC receiving radical RT has dramatically improved recently, owing to better RT technology, the 5-year survival rate of ESCC treated with RT is suboptimal (8). However, the response of ESCC to irradiation (IR) is limited so that numerous patients with ESCC cannot benefit from RT due to radioresistance, which is a major hurdle for successful treatment (9–11). Undoubtedly, exploring molecular markers, which may regulate ESCC radioresistance, to improve clinical outcomes is of primary concern in increasing survival of patients with ESCC.

NRAGE, a neurotrophin-receptor-interacting melanoma antigen-encoding gene homolog, also known as MAGED1 or Dlxin-1, was discovered as a new member of the melanoma antigen family and encodes a cancer-related protein (12, 13). Given its diverse cellular functions, NRAGE is deemed to be greatly crucial in cancer development and progression. Current researchers reported that there were complex and apparently controversial functions on different progression, metastasis, and invasion of tumors (14, 15). Initially, NRAGE was reported as a cancer suppressor gene, which promotes cell apoptosis *via* binding to p75 neurotrophin receptor (P75NTR) (16, 17), Che-1 (18), XIAP-TAK1-TAB1 (19), and UNC5H1 (20) and inhibits proliferation (21) and angiogenesis (22). Contradictorily, functions such as pro-apoptotic gene and growth promotion were slowly discovered (12–14, 23–26). Kodera et al. (25) found that increased NRAGE expression affects the malignant phenotype of hepatocellular carcinoma (HCC) *via* its interaction with apoptosis antagonizing transcription factor (AATF). Zou et al. (24) proved that NRAGE may be a potential biomarker for HCC early diagnosis due to its ability of distinguishing HCC from benign liver disease. Yang et al. (26) reported that an aberrant NRAGE expression in both mRNA and protein levels in ESCC tissues was detected and could induce DNA-damaging chemoresistance by regulating homologous recombination repair.

Originally, our previous studies indicated that NRAGE was significantly overexpressed in the nucleus of ESCC cells with radioresistance (23) and knockdown NRAGE has significantly enhanced radiosensitivity in established radioresistant ESCC cells (14). Scantily, there were only instantaneous intervention at constructed radioresistant EC cells cultured in the 2D level and no correlation with OS of ESCC. Surprisingly, the 3D culture system has a unique superiority in more similar tumor cell growth microenvironment than the 2D *in vitro* system and more short research cycles than the 2D *in vivo* system. This study aimed to confirm the tumor promotor function of NRAGE in ESCC and mechanism that it can accelerate cell growth and survival and induce radioresistance in 2D and 3D culture levels and confer poor prognosis in the clinical setting.

## Methods

### Patient Characteristics

All 44 patients who were clinically and histopathologically diagnosed with primary ESCC based on the WHO criteria, received radical RT through conventional fractionated RT by 6MV X-ray linear accelerator at the Second Hospital of Hebei Medical University from January 2010 to December 2015. The curative effects in the 44 patients were determined 1–3 months later after RT referred to the evaluation standard of esophageal barium swallow. The tissue specimens of the patients were collected, fixed in 4% formalin, and embedded in paraffin. The informed consent of the patients was obtained, and the study was approved by the Ethics Committee of the Second Hospital of Hebei Medical University (No. 20160275). Patient baseline characteristics are shown in **Table S2**.

### 3D Bioprinting of E and E/N Cells

Before printing, the 3D printed workstation (Regenovo Bio-Architect^®^ WS, Hangzhou, China) was sterilized by 75% v/v ethanol and irradiated under UV for 30 min. E and E/N cells (5 × 10^6^) were suspended in 0.2 ml culture medium, followed by the addition of 3 ml of a gelatin–sodium alginate blend (10% gelatin and 3% sodium alginate). Gelatin and sodium alginate were purchased from Sigma (St. Louis, MO, USA). The temperature of 3D-printed platform was set at 8°C measuring 10 × 10 × 1.4 mm in size. After printing, the hydrogels were soaked in 3% calcium chloride for 3 min for a crosslink reaction. Subsequently, the 3D bioprinted scaffolds were incubated at 37°C with 5% CO_2_.

### Live/Dead Staining

The survival rate of newly printed 3D structure E and E/N cells was detected by fluorescent live/dead viability assay kit (KeyGen Biotech, Co., Ltd., Nanjing, China) according to the instructions of the manufacturer. 3D cell-laden constructs were immersed in 1 ml PBS containing 8 μM PI (red, staining dead cells) and 2 μM calcein AM (green, staining living cells) under the conditions of protection from light at room temperature reaction for 15 min and then washed with PBS. Stained cells were imaged using an inverted fluorescence phase contrast microscope (Zeiss, Germany). Live/dead cells were counted in five random fields at ×100 magnification for each sample. The cell death rate was calculated as follows: ratio of cell survival = number of living cells/(number living cells + dead cells) × 100%.

### AlamarBlue

E and E/N cell proliferation in 3D bioprinted hydrogels were tested using an alamarBlue™ cell viability reagent (Invitrogen, USA). 3D-printed scaffolds were washed with PBS, and 500 μl of alamarBlue working solution (alamarBlue: medium = 1:9) was added to each well of a 24-well plate. Then, the 24-well plate was incubated at 37°C and 5% CO2 for 2 h. Then, each 100 μl of working solution was transferred to a 96-well plate, and the absorbances at electron fixation solution at 570 and 600 nm on a multi-function microporous plate detector (bioTek Synergy H1, USA). The scaffolds were cultured for 19–21 days, and the OD values at 570 and 600 nm were detected every 2 days. The cell proliferation ratio calculation was as follows: reduction rate (%) = (A570 − A600 × R) × 100%; R = (A570_control_ − A’570 _control_)/(A600_control_ − A’600 _control_); A570 _control_ and A600_control_, the OD values at 570 and 600 nm of cell-free alamarBlue working solution; A’570 _control_ and A’600_control_, the OD values at 570 and 600 nm of cell-free medium.

### Scanning Electron Microscopy (SEM) Analysis

The 3D-printed scaffolds at 7 and 14 days were fixed with 2.5% glutaraldehyde (Solarbio, Beijing, China) for the night at room temperature and washed three times with PBS for 15 min. The samples were soaked in a series of ethanol solutions (30, 50, 70, 80, 90, 95, and 100%) for 15 min in each solution for dehydration. Subsequently, the scaffolds were dried in the ventilated kitchen. Then, the constructs were coated with platinum (5 nm thickness) and imaged with an Ultra-55 SEM (Zeiss, Germany).

### Cell Culture, Plasmids and Stable Transfection, Realtime PCR, Western Blot Analysis, CCK-8 Assay, IR and Clonogenic Assay, Wound Healing Assay, Transwell Invasion Assay, Flow Cytometry Analysis, γ-H2AX Immunofluorescence Staining, Histology and Immunohistochemistry (IHC)

Experimental analyses were conducted as described previously (14, 27) and detailed in the **Supplementary Materials and Methods**.

### Statistical Analysis

*In vitro* experiments were analyzed by unpaired two-sided Student’s t-test, Welch’s t-test, one-way analysis of variance (ANOVA) or two-way ANOVA. The total and nuclear protein expression of NRAGE and β-catenin in ESCC tissues were analyzed by Mann–Whitney U test. Survival curves were plotted using the Kaplan–Meier method and compared using the log-rank (Mantel–Cox) test. These statistical analyses were conducted by GraphPad Prism 8.0 software. Clinicopathological characteristics of patients with EC following radical RT were analyzed using the chi-squared test. The correlation between NRAGE or β-catenin and clinicopathological features of patients was analyzed using Spearman analysis. The linear correlation between NRAGE nuclear protein and β-catenin nuclear protein were analyzed using the Mantel–Haenszel chi-squared test. Survival data were evaluated using univariate log-rank test and multivariate Cox regression analyses. These data were analyzed using SPSS 25 statistical software (SPSS Inc., Chicago, IL, USA). A *P*-value <0.05 was considered statistically significant.

## Results

### Overexpression of NRAGE Induces Radioresistance of ESCC Cells in 2D Culture

**Figure 1:**
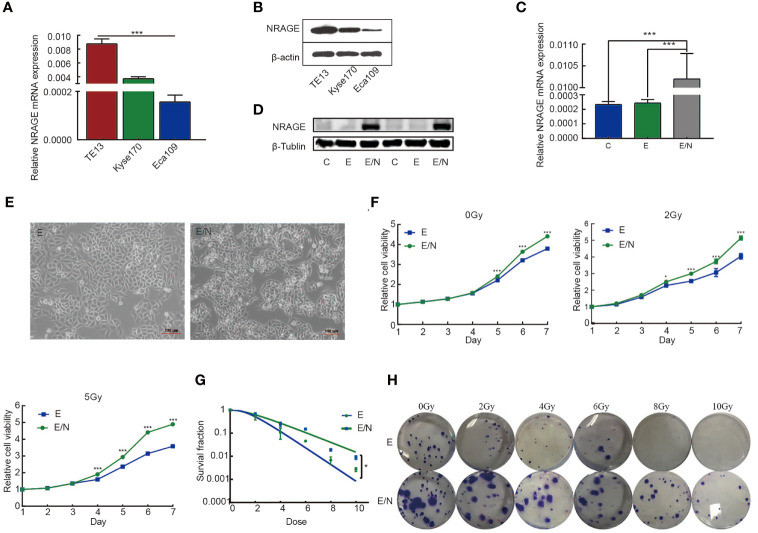
Overexpression of NRAGE enhanced the proliferation and radioresistance of ESCC cells in 2D culture. **(A)** Quantification and analysis of NRAGE mRNA in different ESCC cell lines. **(B)** NRAGE protein levels were analyzed using western blotting in different ESCC cell lines. **(C, D)** Realtime PCR and Western blot assays to determine the overexpression efficiency of transduced Eca109 cells (C: Eca109; E: Eca109-vector; E/N: Eca109-NRAGE); **(E)** cellular morphology compared between E and E/N cells; **(F)** growth curve was detected by CCK8 analysis upon NRAGE stable transfection followed by different irradiation doses with 0, 2 or 5 Gy; **(G)** Dose–response curves were fitted according to the multi-target, single-hit model and analyzed using GraphPad Prism 8.0 software. **(H)** Representative images of colony formation of E and E/N cells after exposure to radiation. All data represented as means ± SD. *p < 0.05 vs. E; ***p < 0.001 vs. E.

Our previous studies indicated that NRAGE was upregulated in ESCC radioresistant cells and extremely likely to be a RT-related critical factor (14, 23, 28). Inadequately, there was lack of direct evidence to confirm the effect of NRAGE on resistance-promoting to IR. To verify the association between NRAGE and ESCC radioresistance, the expression of NRAGE in three types of ESCC cells, TE13, Kyse170, and Eca109, were compared (**Figures 1A, B**). Moreover, Eca109 cells with the least NRAGE expression was selected to stably overexpress NRAGE (**Figures 1C, D**). First, compared with Eca109-vector cells (indicated below as E), we aimed to identify the cellular changes resulting from expression of NRAGE in Eca109 cells (indicated below as E/N). It was visibly different in morphological distinction with more irregular, elongated spindle-shaped cells, and disappearance of polarity (**Figure 1E**). Additionally, cell proliferation and radiosensitivity were tested through CCK-8 and clone formation assay. E/N cell exhibits its super growth ability and radiation-hardened effect (**Figures 1F–H**). Before exposure to IR, both E and E/N cells showed vigorous multiplication without difference during the first 4 days. From the fifth day, E/N cells displayed enhanced proliferation ability. However, in the IR group, the significant difference between them was observed early at the fourth day (E *vs* EN, 0Gy: *p*_5d _<0.0001, *p*_6d _<0.0001, *p*_7d _<0.0001; 2 Gy: *p*_4d_ = 0.0277, *p*_5d _<0.0001, *p*_6d _<0.0001, *p*_7d _<0.0001; 5 Gy: *p*_4d _<0.0001, *p*_5d _<0.0001, *p*_6d _<0.0001, *p*_7d _<0.0001; **Figure 1F**). Moreover, E and E/N cells were exposed to different doses of radiation for colony formation. E/N cells showed relatively higher colony survival rates (*p* = 0.0429) and increased radiobiological parameter, SF2 (E vs EN = 0.518 vs 0.636), D0 (E vs EN = 1.201 vs 2.020), and Dq (E *vs* EN = 1.492 *vs* 1.530), under a series of doses of 0, 2, 4, 6, 8, and 10 Gy (**Table S1**; **Figures 1G, H**). These results suggest that NRAGE overexpression induces radioresistance of ESCC cells in 2D culture.

### Overexpression of NRAGE Regulates Cell Migration, Invasion, Cell Cycle Progression and Apoptosis After IR in 2D Culture

**Figure 2:**
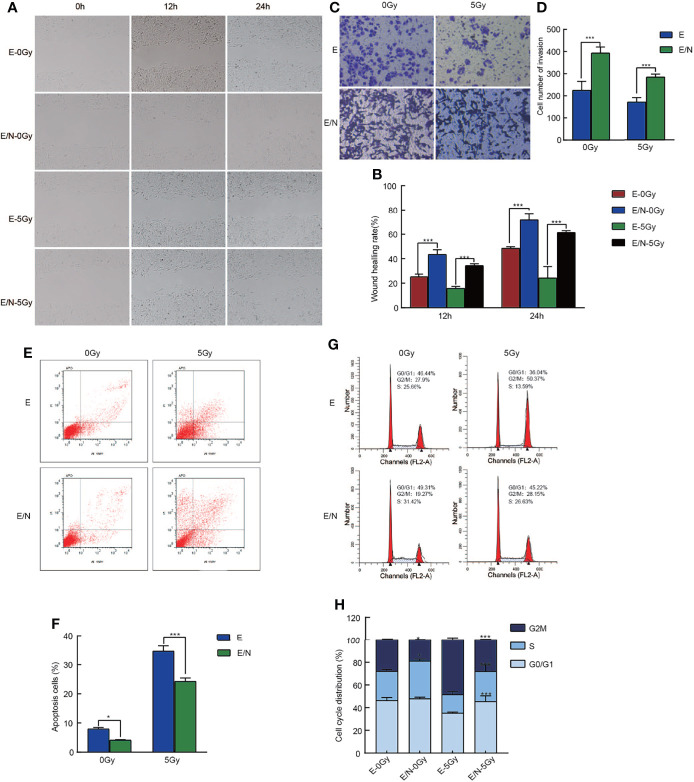
Overexpression of NRAGE inhibits cell migration, invasion, cell cycle progression and apoptosis after IR in 2D culture. **(A)** Wound Healing assay was applied to test the migration ability of E and E/N with or without 5 Gy irradiation (magnification: ×200); **(B)** Quantitative assessment of wound-healing rate at the different times (12 and 24 h); **(C)** Matrigel invasion assay was applied to compare E and E/N cells for invasion ability with or without 5 Gy irradiation (magnification: ×200); **(D)** Quantitative assessment of the number of invasion cells. *P < 0.05 vs. E; **P < 0.01 vs. E; ***P < 0.005 vs. E; **(E)** Annexin V-FITC/PI staining was applied to test the apoptotic rates of E and E/N cells with or without 5 Gy IR by flow cytometry; **(F)** Quantitative assessment of the apoptotic rates; **(G)** Propidium iodide stain was applied to test cell cycle of E and E/N cells with or without 5 Gy IR by flow cytometric; **(H)** Cell cycle distributions were analyzed using GraphPad Prism 8.0 software; All data represented as means ± SD. *p < 0.05 vs. E; ***p < 0.001 vs. E.

To further define the mechanism of NRAGE in ESCC radioresistance, cell migration, invasion, cell cycle progression, and apoptosis in E/N cells were analyzed in addition to the detection of proliferation. Wound healing assays were performed in E and E/N cells with or without 5 Gy X-ray radiation and then imaged at 12 and 24 h. Results showed that ESCC cells with upregulated NRAGE had significantly faster migration ratio than E cells, especially after IR (**Figures 2A, B**). Transwell assays also showed that the number of invasion cells through the membrane regardless of the presence of IR was significantly larger in E/N cells (**Figures 2C, D**). It revealed that NRAGE may enhance invasion and migration of ESCC cells after IR led to more resistive effect to IR. Cell apoptosis assays showed that the rate of spontaneous apoptosis in E/N cells was significantly decreased (E vs E/N, 7.99% ± 0.50% vs 4.16% ± 0.15%, *p* = 0.0057). After RT, E/N cells had lower apoptosis than E cells (E vs E/N, 24.29% ± 1.12% vs 34.63% ± 1.83%, *p <*0.0001) (**Figures 2E, F**). Furthermore, we analyzed the cell cycle progression of E and E/N cells with or without 5 Gy IR (**Figures 2G, H**). It was found that, before IR exposure, NRAGE overexpression was associated with an increased percentage of cells in the S phase (33.23 ± 1.78 vs 25.69 ± 1.70, *p* = 0.01), the most radioresistant cell stage, and a lower ratio in the most radiosensitive cell stage G2/M (18.87 ± 0.46 vs 27.91 ± 0.81, *p* = 0.0018). After treatment with 5 Gy IR, cell cycle distributions were rearranged to a greater extent with more arrested cells in the S phase (26.46 ± 5.61 vs 16.27 ± 2.71, *p* = 0.0005) and G0/G1 phase (45.50 ± 4.95 vs 35.21 ± 0.96, *p* = 0.0004) and downregulated cells in the G2/M phase (28.04 ± 0.67 vs 48.52 ± 1.77, *p <*0.0001). These revealed that NRAGE overexpression could reduce cell apoptosis and change cell cycle division of ESCC, affecting cellular radioresistance.

### NRAGE Overexpression Activates Canonical Wnt Signaling Pathway in ESCC Cells With 2D Culture

**Figure 3:**
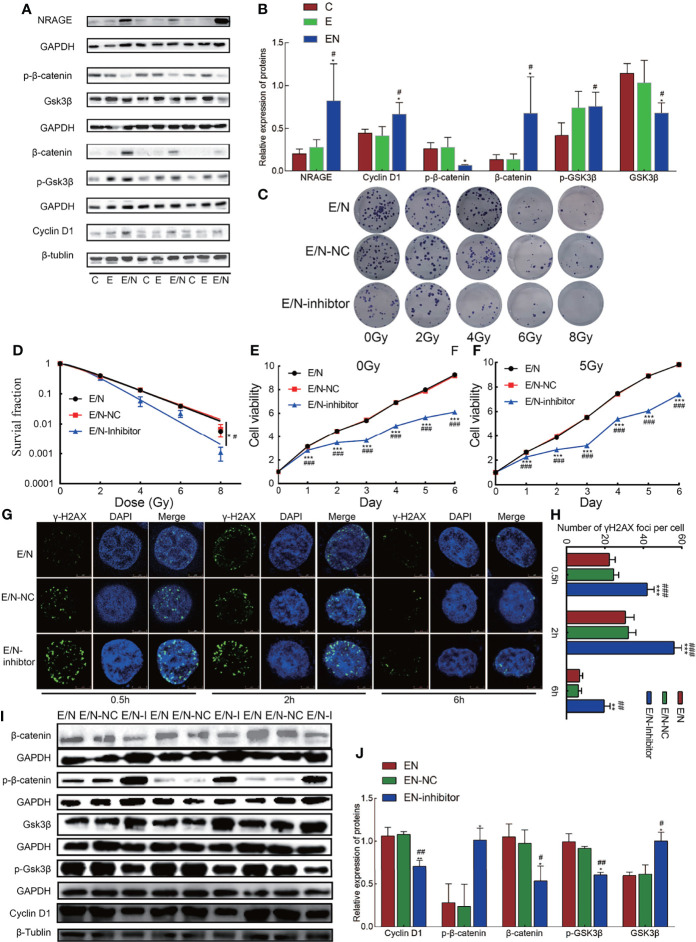
Overexpression of NRAGE activates canonical Wnt signaling pathways in ESCC cells with 2D culture. **(A)** The expression of Wnt/β-catenin signaling pathway-related proteins was determined using western blotting. **(B)** Quantitative assessment of related proteins in Wnt/β-catenin signaling pathway. All data represented as means ± SD. *p < 0.05 vs. E; **p < 0.01 vs. E; ***p < 0.001 vs. E, ^#^ *p <* 0.05 vs. C; ^##^ *p <* 0.01 vs. C; ^###^ *p <* 0.001 vs. C. **(C)** Representative images of colony formation of E/N cells after exposure to radiation with or without FH535. **(D)** Dose–response curves were fitted according to the multi-target, single-hit model and analyzed using GraphPad Prism 8.0 software. **(E, F)** growth curve was detected by CCK-8 analysis in EN cells with or without FH535 by different irradiation doses with 0 and 5 Gy. **(G, H)** The formation of γ-H2AX foci at 0.5, 2, 6 after 5 Gy irradiation in EN cells with or without FH535. **(I, J)** Western blotting analysis the expression of Wnt/β-catenin signaling pathway-related proteins with or without FH535. All data represented as means ± SD. *p < 0.05 vs. EN; **p < 0.01 vs. EN; ^#^ *p <* 0.05 vs. EN-NC; ^##^ *p <* 0.01 vs. EN-NC.

Previous studies indicated that NRAGE had a potential association with β-catenin in the formation of radioresistance in ESCC (14), so we detected protein expression in canonical Wnt signaling pathway, namely, β-catenin, phosphorylation of β-catenin (p-β-catenin), Gsk-3β, phosphorylation of Gsk-3β (p-Gsk-3β), and CyclinD1. Compared with E, an increase in β-catenin (*p*= *0.037*) level, followed by increased trend of p-Gsk-3β levels (*p* = *0.917*), led to the increased expression of cyclin D1 (*p* = 0.023), a targeting gene of β-catenin in E/N cells, while downregulation of p-β-catenin (*p* = 0.037) and Gsk-3β (*p* = *0.049*) was detected (**Figures 3A, B**). To further investigate the role of the Wnt/β-catenin signaling pathway in radioresistance of ESCC regulated by NRAGE, FH535 (HY-15721, MCE, USA), a reversible inhibitor of the Wnt pathway, was used. After treated with FH535, E/N cells were tested the radioresistance and proliferation by clonogenic and cck-8 assay. The results showed that E/N cell treated with inhibitor had significant decline on both colony survival (E/N vs E/N-inhibitor: *p* = 0.0139; E/N-NC vs E/N-inhibitor, *p* = 0.0442, **Figures 3C, D**) and proliferation rates (**Figures 3E, F**). In addition, γ-H2AX Immunofluorescence experiment exhibited that E/N cells with FH535 had a remarkable higher formation of γ-H2AX foci after irradiation than both E/N and E/N-NC cells at 0.5 h (E/N vs E/N-inhibitor, 22.33 ± 3.06 vs 42 ± 3.60, *p <*0.0001; E/N-NC vs E/N-inhibitor, 24.67 ± 2.52 vs 42 ± 3.60, *p* = 0.0003), 2 h (E/N vs E/N-inhibitor, 30.67 ± 4.51 vs 56 ± 4.00, *p <*0.0001; E/N-NC vs E/N-inhibitor, 32.33 ± 3.79 vs 56 ± 4.00, *p <*0.0001), 6 h (E/N vs E/N-inhibitor, 6.83 ± 1.61 vs 19.67 ± 3.06, *p* = 0.003; E/N-NC vs E/N-inhibitor, 6.33 ± 1.53 vs 19.67 ± 3.06, *p* = 0.0023) (**Figures 3G, H**). Western blot results demonstrated that CyclinD1 (E/N vs E/N-inhibitor, *p* = 0.001; E/N-NC vs E/N-inhibitor, *p* <0.001), β-catenin (E/N vs E/N-inhibitor, *p* = 0.008; E/N-NC vs E/N-inhibitor, *p* = 0.015) and p-Gsk-3β (E/N vs E/N-inhibitor, *p* = 0.030; E/N-NC vs E/N-inhibitor, *p = 0.008*) decreased, while p-β-catenin (E/N vs E/N-inhibitor, *p* = 0.074; E/N-NC vs E/N-inhibitor, *p* = 0.025) and Gsk-3β (E/N vs E/N-inhibitor, *p* = 0.037; E/N-NC vs E/N-inhibitor, *p* = 0.031) slightly increased after FH535 treatment (**Figures 3I, J**) in E/N cells. These data indicated that after NRAGE overexpression, the canonical Wnt signaling pathway was overall activated, which may be a switch-induced radioresistance. Also, the inhibitor of Wnt/β-catenin signaling pathway, FH535, could reverse the enhanced radioresistance induced by NRAGE overexpression.

### 3D Bioprinted ESCC Cell-Laden System Cultured *In Vitro*

**Figure 4:**
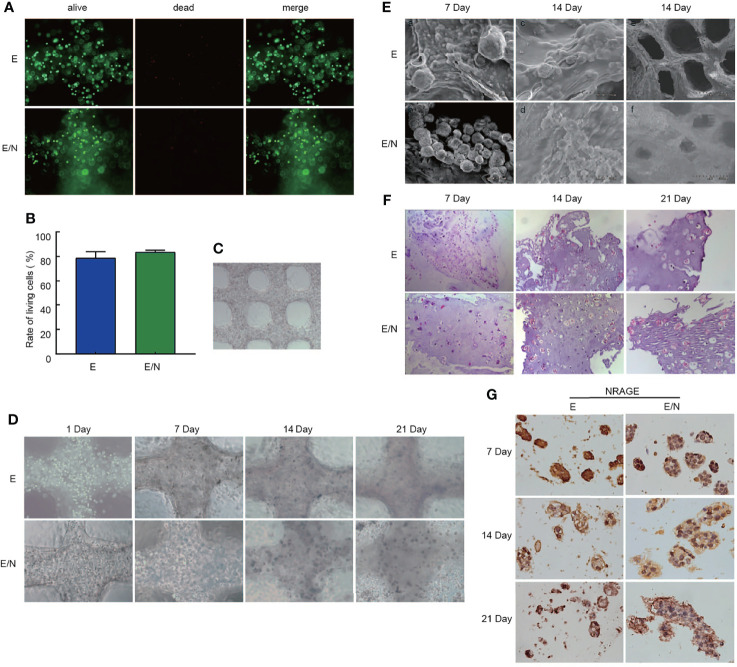
3D bioprinted ESCC cell-laden system cultured *in vitro*. **(A)** Live/dead staining for cell viability after printing, where live cells are stained in green and dead cells in red. **(B)** Cell viability of E and E/N cells after printing. **(C)** 3D bioprinted E and E/N cells at day 1 of culturing. **(D)** optical microscopy images. **(E)** SEM images. **(F)** Hematoxylin–eosin staining: 3D bioprinted E and E/N cells cultured *in vitro* for 7, 14, and 21 d **(G)** Immunohistochemistry of 3D bioprinted E and E/N cells: NRAGE expression at 7, 14, and 21 d in culture. Scale bars: **(A, D, F)** 100 μm; **(C)** 200 μm; **(E)** a–d 50 μm, e–f 500 μm; **(G)** 50 μm.

To the best of our knowledge, there is considerable difference on the cancer cell morphology genetic profile and tumoral heterogeneity in 2D cultures. To focus more on the tumor cell growth environment and microenvironment *in vitro*, we selected culture cells in the 3D bioprinting system to identify the function of NRAGE in radioresistance of ESCC cells. A gelatin–alginate blend (10% gelatin and 3% sodium alginate) was used as the 3D bioprinted material. Hydrogel seeded with E and E/N cells were extruded at variable pressure (0.31 MPa), needle type (cylindrical), and needle diameter (340 µm) to study cell characteristics directly after printing. Extruded gelatin–alginate blend was stained with live/dead dye and imaged (**Figures 4A, B**). Most cells remained viable (green), and only a small number of dead cells (red) were observed. Subsequently, the result of the analyses showed that dead or live cells were counted to quantify cell survival at >80% and E/N cell-laden 3D-scaffolds exhibited stronger survivability. After printing, images were obtained, followed by crosslinking in 3% CaCl_2_ and incubation at 37°C to allow the gelatin to leach out. The cell-laden 3D-scaffolds had a grid-like structure arranged in multiple layers, and cells were uniformly distributed in porous scaffolds with tight order, exhibiting good cytocompatibility (**Figure 4C**). At the first week, the printed scaffolds did not display obvious proliferation. Then, the cell growth rate accelerated slowly over time. After 3 weeks, cells began to grow into spheroids and pushed the surrounding hydrogels aside to occupy a larger space. Especially for E/N cell-laden hydrogels, the phenomena were highlighted. It was extremely biomimetic to the solid tumor growth *in vivo*. SEM observations revealed the spheroids bulged out over the scaffolds surface, and the trace of cells squeezed the surrounding hydrogel, which showed that E/N cell-laden scaffolds were apt to spheroiding. It was also suggested that E/N cells in the 3D group have a significantly higher secretion of growth hormone than E cells, and the difference gradually became pronounced over time (**Figure 4E**). After culture for 7 days, as shown in HE staining, individual cells scattered in printed scaffolds were observed (**Figure 4F**). During the culture period, natural gelatin began to degrade gradually *via* hydrolysis in the culture medium and then provided space for cells proliferating in clusters at 14 and 21 days. Furthermore, more and larger cell clusters were observed in E/N cell-laden 3D scaffolds. **Figure 4G** shows that both positive expressions of NRAGE were observed at E-3D and E/N-3D cells, while NRAGE in E/N cells in the 3D culture system had a distinct positive staining in nuclear cells with larger cluster. These results implied that cells with NRAGE overexpression in the 3D culture system are more suitable for survival and cloud growth, which hinted that ESCC cells with NRAGE overexpression exerted greater adaptability to survive and multiply.

### NRAGE Overexpression Enhanced the Proliferation and Radioresistance of ESCC Cells in 3D Bioprinted Hydrogels

**Figure 5:**
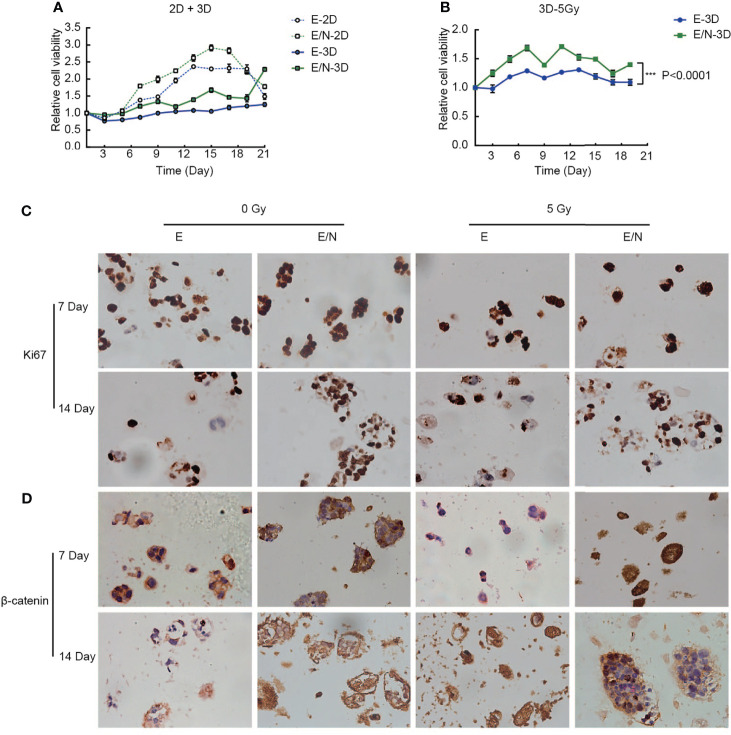
Overexpression of NRAGE enhanced the proliferation and radioresistance of ESCC cells in 3D bioprinted hydrogels. **(A)** Comparing cell proliferation between E and E/N in 2D and 3D by alamarBlue assays; **(B)** Cell proliferation was tested by immunohistochemistry of 3D bioprinted E and E/N cells: Ki-67 expression at 7,14 d in culture with or without IR; **(C)** cell viability between E and E/N in 3D after 5 Gy IR by alamarBlue assays; **(D)** Immunohistochemistry of 3D bioprinted E and E/N cells: β-catenin expression at 7, 14, and 21 d in culture with or without IR. Scale bars: **(B, D)** 50 μm, all data represented as means ± SD. ***p < 0.001 vs. E.

The obvious advancement and characteristic of the 3D-printed model showed more similar growth environment and microenvironment *in vivo*. To identify the effect of NRAGE overexpression in ESCC cells cultured in the 3D-printed model on proliferation of tumor cells, alamarBlue assays were selected to compare cell viability between 3D-printed and 2D groups. As shown in **Figure 5A**, regardless of the culture condition (2D or 3D), E/N cells had considerably higher survival percentages than E cells. More interestingly, there was a faster proliferation rate in E/N cells in the 2D group in the first 20 days, whereas the 3D-printed group showed a significantly higher proliferation rate of cells after 20 days. Similarly, an apparent trend that E cells in the 3D-printed group would proliferate faster than those in the 2D group after 21 days was observed (**Figure 5A**). Moreover, the difference in responses to IR between E and E/N cells in the 3D-printed model was identified by alamarBlue assays after 5 Gy IR. Evidently, E/N-3D cells had an absolute dominance of survival on resistance to IR compared with E-3D cells (**Figure 5B**). Deeply, the protein expression of Ki-67, a marker for cell proliferation activity, between E-3D and E/N-3D cells with or without 5 Gy IR, was evaluated by IHC, and both of them had a positive expression in relative individual cells scattered in hydrogel at 7 days. However, after 14 days, more positive staining in larger clusters in E/N-3D cells appeared. Furthermore, this different trend was also found after 5 Gy IR (**Figure 5C**). To verify whether the β-catenin expression change in ESCC cells with NRAGE overexpression was consistent from 2D to 3D groups, IHC staining was performed. Similarly, in the first 7 days, both E-3D and E/N-3D cells had a higher β-catenin expression levels with larger cell clusters. After culture for 14 days, larger E/N-3D cell clusters were stained positively by β-catenin antibody compared with those in E cells. Unsurprisingly, there was a more obvious distinction between groups after 5 Gy IR (**Figure 5D**). The results confirmed further that accelerated NRAGE expression in ESCC cells activate β-catenin expression to regulate radiosensitivity.

### NRAGE is Upregulated in Patient Samples With EC Following Radical RT and Correlated With Poor Prognosis

**Figure 6:**
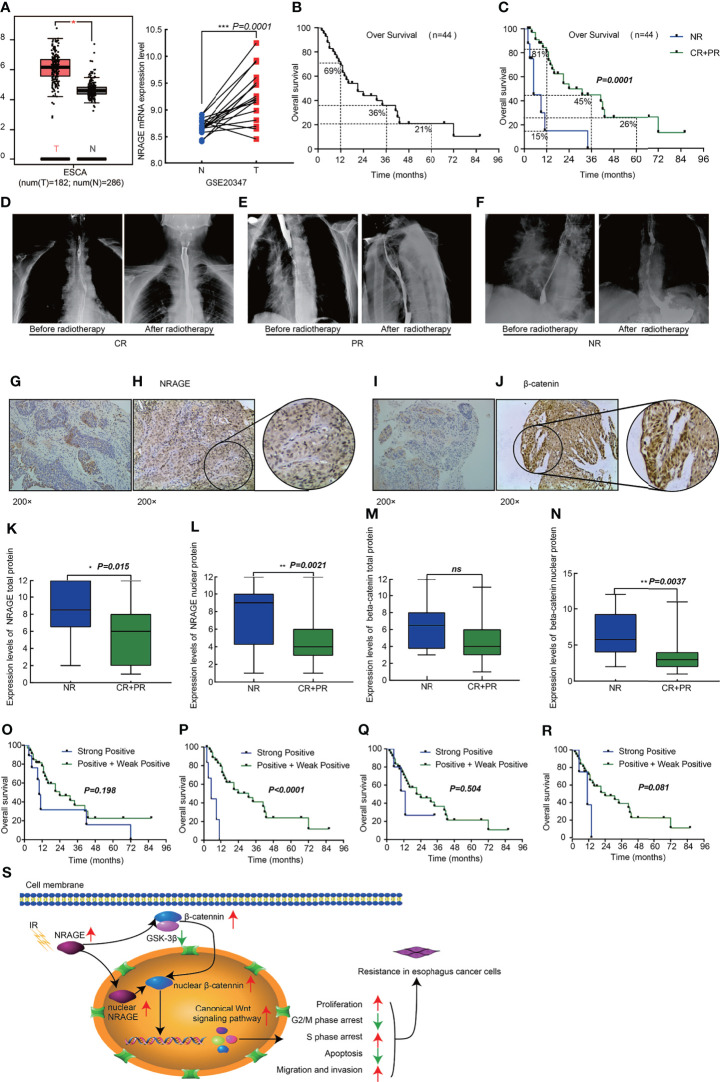
High expression nuclear NRAGE in patient samples with ESCC following radical radiotherapy correlates with poor survival. **(A)** NRAGE Expression in esophageal cancer tissues and normal control esophageal tissues from the TCGA (left, ESCA) and the GSE20347 (Right, ESCC) data; **(B)** Kaplan–Meier overall survival curves for all 44 patients with esophagus cancer; **(C)** Kaplan–Meier overall survival curves for all 44 patients with esophagus cancer stratified by NR and CR + PR; **(D–F)** Images of radiotherapeutic short-term effects, CR **(D)**, PR **(E)**, NR **(F)**; **(G, H)** Representative images of NRAGE negative **(G)** and positive **(H)** expression; **(I, J)** Representative images of β-catenin negative **(I)** and positive **(J)** expression; **(K, L)** Comparison of NRAGE total **(K)** and nuclear **(L)** protein expression between NR group and CR + PR group; **(M, N)** Comparison of β-catenin total **(M)** and nuclear **(N)** protein expression between NR group and CR + PR group; **(O, P)** Kaplan–Meier overall survival curves for all 44 patients with esophagus cancer stratified by strong positive and weak positive+positive expression of NRAGE total **(O)** and nuclear **(P)** protein; **(Q, R)** Kaplan–Meier overall survival curves for all 44 patients with esophagus cancer stratified by strong positive and weak positive + positive expression of β-catenin total **(Q)** and nuclear **(R)** protein; **(S)** Schematic illustration depicting the NRAGE associations with cancer proliferation, apoptosis, cell cycle and invasive migration that induce resistance to radiation. *p < 0.05; **p < 0.01; ***p < 0.001; ns, no significance.

We analyzed NRAGE expression in public database, and found that it was upregulated both in ESCA samples (182 cases) compared with adjacent normal tissue samples (286 cases) (*p <*0.05) (match TCGA normal and GTEx data, http://gepia.cancer-pku.cn/) and in ESCC samples compared with paired Paracancerous tissue from the GSE20347 dataset (*p* = 0.0001, https://www.ncbi.nlm.nih.gov/geo/) (**Figure 6A**). Additionally, to thoroughly explore the role of NRAGE on radioresistance of ESCC and relationship with β-catenin, we further analyzed the expression of NRAGE and β-catenin in a total of 44 paraffin-embedded, ESCC tumor tissues receiving definitive RT (**Table S2**). As shown in **Figure 6B**, the 1-, 3-, and 5-year overall survival rates of 44 patients were 69, 36, and 21%, respectively (**Figure 6B**). According to the evaluation criteria of RT curative effect, 36 patients were classified in the efficacy group (complete response, CR, 26 patients, and partial response, PR, 9 patients) and 8 patients were classified in the inefficacy group (No response, NR, 8 cases) (**Figures 6D–F**). There were statistically significant differences between the two groups in the 1-, 3-, and 5-year OS rates: 81, 45, and 26% for the efficacy group and only 15% of 1-year OS rate in the inefficacy group were achieved (*p = 0.0001*) (**Figure 6C**). Compared to the inefficacy group in which NRAGE and β-catenin were expressed at low levels (**Figures 6G, I**), NRAGE and β-catenin were overexpressed, especially for positive nuclear expression, in efficacy group specimens (**Figures 6H, J**). According to the analysis of the relationship between staining score and short-term effect of RT, NRAGE protein expression was dramatically upregulated in the NR group tumor tissues compared with the efficacy group (CR + PR)(P = 0.015) (**Figure 6K**). Unsurprisingly, more NRAGE nuclear protein expression were detected in NR group (*p* = 0.0021) (**Figure 6L**). Additionally, there was higher β-catenin total protein expression in the NR group than in the efficacy group (*p* = 0.081) (**Figure 6M**). However, the difference in β-catenin nuclear protein expression between the two groups was significant (*p* = 0.0037) (**Figure 6N**).

Routinely, we analyzed the association between NRAGE total/nuclear protein or β-catenin total/nuclear protein expressions and clinicopathological features of 44 patients with ESCC by Spearman analysis. It was revealed that the expression of NRAGE total protein, especially for NRAGE nuclear protein, was strongly associated with curative efficacy (*p =* 0.0023, *p =* 0.006). However, regardless of NRAGE total protein or NRAGE nuclear protein, there was no association with age (*p =* 0.656, *p =* 0.277), gender (*p =* 0.734, *p* = 0.277), clinical stage (*p =* 0.932, *p* = 0.759), tumor size (*p =* 0.121, *p* = 0.488), LNM (*p =* 0.153, *p* = 0.148), synchronous chemotherapy (*p =* 0.906, *p* = 0.862), and events (*p* = 0.135, *p =* 0.528) (**Table 1**). As shown in **Table 1**, no correlation between β-catenin expression and age (*p =* 0.288, *p =* 0.231), sex (*p =* 1.000, *p =* 0.358), clinical stage (*p* = 0.824, *p =* 0.986), tumor size (*p =* 0.168, *p =* 0.263), LNM (*p =* 0.221, *p =* 0.587), synchronous chemotherapy (*p =* 0.099, *p* = 0.459), and events (*p =* 0.754, *p =* 0.296) was found. A significant correlation could not be found between β-catenin total protein expression and clinicopathological features (*p =* 0.143), but a strong association between β-catenin nuclear protein expression and curative efficacy was observed (*p =* 0.006). Kaplan–Meier survival curves exhibited no association between OS in definitive RT and NRAGE or β-catenin total protein expression (**Figures 6O, Q**, *p =* 0.198, *p =* 0.504), but a strong positive NRAGE nuclear protein expression was significantly shorter than those with positive and weak positive NRAGE expression (**Figure 6P**, *p <*0.0001). Additionally, there was a correlated trend between β-catenin nuclear protein expression and OS (**Figure 6R**, *p =* 0.081). Moreover, we analyzed the association between NRAGE and β-catenin nuclear protein expressions and found their linear correlation trend (cor = 0.291, *p* = 0.055) (**Table S3**). These results indicate that NRAGE expression, especially NRAGE nuclear expression, in patients with ESCC receiving radical RT was correlated with poor survival and may be linked to heightened β-catenin nuclear accumulation. Furthermore, univariate, and multivariate analyses were used to determine whether NRAGE could be a risk factor in patients with ESCC receiving radical RT. Log-rank test in the univariate analysis showed that synchronous chemotherapy (*p* = 0.037), curative efficacy (*p* = 0.000), and strong positive NRAGE nuclear protein expression (*p* = 0.000) were associated with a significantly increased risk of death in patients with ESCC receiving radical RT (**Table 2**). Multivariate Cox regression analysis revealed that NRAGE nuclear protein could be a factor for predicting poor survival when it has strong positive expression (*HR =* 14.536, *p =* 0.000). Synchronously, clinical stage (*HR =* 2.995, *p =* 0.024) and synchronous chemotherapy (*HR =* 0.354, *p =* 0.019) were included as factors (**Table 2**). Collectively, all these indicated that NRAGE overexpression occurred during nuclear translocation after IR and stimulated β-catenin expression in the cytoplasm to increase the nuclear localization of β-catenin, which activated the Wnt/β-catenin signaling pathway and then induced the radioresistance in ESCC. A flowchart of the possible mechanism is shown in **Figure 6S**.

**Table 1:** Spearman analysis of correlation among NRAGE, β-catenin and clinicopathological features.

Variables	Group	NRAGE expression		NRAGE nuclear expression		β-catenin expression		β-catenin nuclear expression	
		Spearman correlation	*P*-value	Spearman correlation	*P*-value	Spearman correlation	*P*-value	Spearman correlation	*P*-value
**Age (y)**	≥60	−0.069	0.656	−0.168	0.277	−0.164	0.288	0.184	0.231
	<60								
**Gender**	Male	0.053	0.734	0.168	0.277	0.000	1.000	0.142	0.358
	Female								
**Clinical Stage**	I–II	−0.013	0.932	0.048	0.759	−0.034	0.824	−0.003	0.986
	III–IV								
**Tumor size**	≥5 cm	−0.237	0.121	−0.107	0.488	−0.212	0.168	−0.172	0.263
	<5 cm								
**lymph nodes metastasis (LNM)**	Yes	−0.219	0.153	−0.222	0.148	−0.188	0.221	0.084	0.587
	No								
**Synchronous chemotherapy**	Yes	0.018	0.906	−0.027	0.862	0.252	0.099	−0.115	0.459
	No								
**Curative efficacy**	effectivity	−0.342	***0.023***	−0.405	***0.006***	−0.225	0.143	−0.41	**0.006**
	inefficacy								
**Events**	Censored	0.229	0.135	0.098	0.528	0.049	0.754	0.161	0.296
	Dead								

The italic font and bold values represents there was a significance.

**Table 2:** Univariate and Multivariate analysis of various prognostic parameters in patients with ESCC following Radical radiotherapy.

Univariate analysis					Multivariate analysis			
Variable	OS (m)		Log Rank χ^2^	*P-*value	Variable	HR	95% CI	*P-*value
	Median (95% CI)							
**Age (y)**			1.314	0.252	**clinical stage**	2.995	(1.154–7.775)	0.024
**≥60**	15	(5.734–24.266)						
**<60**	34	(7.584–60.416)						
**Gender**			0.895	0.344	**Synchronous chemotherapy**	0.354	(0.149–0.843)	0.019
**Male**	16	(0–42.659)						
**Female**	21	(8.291–33.709)						
**Clinical Stage**			3.222	0.073	**Expression of NRAGE nuclear**	14.536	(3.847–54.928)	0.000
**I–II**	31	(6.170–55.830)						
**III–IV**	15	(8.452–21.548)						
**Tumor size**			0.145	0.704				
**≥5 cm**	21	(10.280–31.720)						
**<5 cm**	16	(0–49.001)						
**lymph nodes metastasis (LNM)**			0.001	0.973				
**Yes**	21	(6.566–35.434)						
**No**	34	(1.442–66.558)						
**Synchronous chemotherapy**			4.366	***0.037***				
**Yes**	41	(13.456–68.544)						
**No**	14	(9.020–18.980)						
**Curative efficacy**			14.831	***0.000***				
**effectivity**	24	(7.702–40.298)						
**inefficacy**	5	(2.540–7.460)						
**Expression of NRAGE**			1.660	0.198				
**Weak positive + Positive**	24	(8.038–39.962)						
**Strongly positive**	10	(7.690–12.310)						
**Expression of NRAGE nuclear**			23.831	***0.000***				
**Weak positive + Positive**	34	(14.792–47.208)						
**Strongly positive**	5	(1.080–8.920)						
**Expression of β-catenin**								
**Weak positive + Positive**	21	(4.322–37.678)	0.446	0.504				
**Strongly positive**	13	(6.363–19.637)						
**Expression of β-catenin nuclear**			3.040	0.081				
**Weak positive + Positive**	24	(6.734–41.266)						
**Strongly positive**	11	(2.018–19.982)						

The italic font and bold values represents there was a significance.

## Discussion

EC is one of the most common primary malignancies with high mortality, and mainly in ESCC in China. RT is one of the primary therapeutic modalities in patients with ESCC. The existence of radioresistance is a major limit to achieve long-term survival in ESCC, which has been linked to an increased likelihood of recurrence and distant metastasis (29–32).

NRAGE has complicated and contradictory functions. It encodes an 86-KDa protein and is a member of the Type II MAGE family, comprising 778 amino acids (aa), of which the MAGE homology domain is common for MAGE family members and the interspersed repeat domain (IRD) is relatively unique to NRAGE for no homology to any public specific protein. These features imply that there are both uniform and specific characteristics of NRAGE compared with other members of the MAGE family (33–36). Growing evidence confirmed that NRAGE functions as a transcriptional regulator mediating multiple signaling pathways from apoptosis (16, 18–20), cell differentiation (37–39), cell cycle distribution (26), cell adhesion (40), and angiogenesis (22). In contrast, NRAGE could promote cell apoptosis through interaction with P75NTR (16), Che-1 (18), XIAP-TAK1-TAB1 (19), and UNC5H1 (20). However, Kumar et al. (41) initially found the anti-apoptosis role of NRAGE, in which NRAGE had carried out anoikis resistance after it transferred into the nucleus and coacted with TBX2. Moreover, NRAGE also exerted cell differentiation functions through activating the transcriptional function of Dlx5 (37), downregulating TrkA (38), and been reducing by Praja1 (39). Furthermore, as an important adaptor, NRAGE could interact with PCNA to promote anti-apoptosis, accelerate cell growth, and change cell cycle distribution (26). Additionally, NRAGE could regulate cell adhesion by participating in epithelial–mesenchymal transformation (EMT) activity (40) and angiogenesis by interfering with HIF-1-dependent gene expression (22). Mitsuro et al. (35) reported NRAGE’s carcinogenic role that is the knockdown of NRAGE could reduce proliferation, migration, and invasion in gastric cancer cells, which was positively correlated with AATF. Generally, the above mentioned studies indicate that NRAGE, as a molecular bridge, exerts complex and contradictory functions as either an inhibitor or promoter depending on different cell types.

Innovatively, our team provided a new insight for NRAGE into the ability of pro-radioresistance that NRAGE was unexpectedly overexpressed in radioresistant ESCC cells based on gene microarray analysis and experiment verification. It was shown that NRAGE had a growing trend following enhanced IR dose and time (23). Another study of our team determined whether NRAGE subcellular localization alteration led to radioresistance and may be related to the occurrence of EMT in ESCC (40). Subsequently, our study verified that NRAGE was upregulated in constructed radioresistant cells from ESCC cells TE13 and Eca109 and participated in the information of radioresistance in ESCC (14). Clinical sample detection revealed that there was high expression of NRAGE in patients with ESCC in the invalid group based on short-term efficacy evaluation treated with definitive RT. Inadequately, the abovementioned contents were obtained in 2D level, and the interference measure for NRAGE was transitory RNA interference (RNAi). In addition, the relationship between NRAGE expression and OS of the patients was not analyzed. The evidence that NRAGE overexpression was related to poor prognosis of patients with ESCC treated by radical RT was relatively weak.

In this study, we selected Eca109 cells to artificially stably overexpress NRAGE, in which NRAGE expression was at a related lower level compared with those in TE13 and Kyse170 cells (**Figures 1A–D**). After interference, accelerated growth speed, degressive apoptosis rate, more malignant migration and invasion, and redistributed cell cycle led to the accumulation of IR resistance in Eca109 cells with NRAGE overexpression (**Figures 1F–H**, **2A–H**). Moreover, the experiments in the cell level on the mechanism of NRAGE involved in radioresistance in ESCC cells were performed at both 2D and 3D levels. It is a truism that 3D printing technology has high precision and fast building speed, which can not only improve and more fully simulate the natural microenvironment *in vivo* but also shorten the research cycle compared with the mouse model (42–45). In the 3D system, we printed the 3D E-cells and E/N cell models using a gelatin–alginate blend (10% gelatin and 3% sodium alginate). Through observing model morphology by brightfield and SEM, the 3D E/N cell model showed more powerful spheroiding capacity (**Figures 4D, E**). From the results of HE staining, alamarBlue detection, and ki-67 IHC staining, more strong proliferation ability and resistance to IR were apparent (**Figures 4F**, **5A–C**). As for the mechanism of NRAGE participating in the information of radioresistance in ESCC cells, the Western blot analysis results on the total and phosphorylated β-catenin protein and total and phosphorylated Gsk-3β protein and β-catenin IHC stain results of 3D-cells scaffolds demonstrated that NRAGE may trigger β-catenin nuclear protein accumulation and then activate the canonical Wnt signaling pathways to motivate cancer-promoting activities (**Figures 3A, B**). Additionally, after treated with FH535 in E/N cells, the canonical Wnt signaling pathways genes Cyclin D1, β-catenin, and p-Gsk-3β were downregulated specifically, while p-β-catenin and Gsk-3β were upregulated, indicating the inhibition of the Wnt/β-catenin pathway (**Figures 3I, J**). Subsequently, we tested whether FH535 could reverse the radioresistance and proliferation by a colony forming assay and CCK-8 analysis. The conjecture was proved following decreased colony survival and proliferation rates in FH535 treated group (**Figures 3C–F**). Furthermore, the increase formation of γ-H2AX foci, a sensitive indicator of DNA repair, in E/N cells after added FH535, indicates poorer DNA damage repair ability. Altogether, it was suggested that FH535 could potentially act as a radiosensitizer for E/N cells.

β-catenin, a core member of the canonical Wnt signaling pathway, was strongly linked to EC progression, metastasis, and invasion (46–49). In off state of canonical Wnt signaling pathways, β-catenin is mainly expressed at the cytoplasm, and Gsk-3β inhibits tumor growth by degrading β-catenin. However, in the on state, Gsk-3β was inactivated through phosphorylation, and then β-catenin is accumulated in the cytoplasm so as to increase the nuclear localization of β-catenin, activating downstream target genes and modulating the behavior of tumor cells. Prominently, numerous investigations showed a close-knit relationship between radioresistance and β-catenin in EC (14, 50–56). Che et al. (53) used fractionate IR to acquire radioresistant EC cells Eca109R50Gy and tested the aberrant expression of β-catenin. When treated with a COX-2 inhibitor, downregulation of β-catenin and enhanced radiosensitivity were observed. In 2014, Su et al. screened three mircroRNAs (mir-301a, mir-131, and mir-18b) based on human miRNA microarray results reserved on Public DataBase on radioresistant ESCC cell line KYSE-150R and its parental KYSE-150 and subsequent real-time qPCR verification. Target gene prediction revealed that wnt1 was a potential target gene of mir-301a and overexpressed in KYSE-150R (54). Their subsequent study confirmed that microRNA−301a could increase the radiosensitivity and restrain the migration of ESCC cells with radioresistance through affecting canonical Wnt signaling pathways (50). Another study of the team showed EMT phenotypes and acquisition of radioresistance in EC cells were related to activation of the canonical Wnt signaling pathway. Moreover, a type of this pathway inhibitor, FH535, can reverse the abovementioned phenomenon (51). Previously, our team presented the correlation between NRAGE and β-catenin only in the cell level (14). Unfortunately, many studies were short of clinical relevance.

Profoundly, in the present study, with further analysis, we found the close-knit pertinence between NRAGE and β-catenin in the clinical setting, in which NRAGE protein expression level, especially for NRAGE nuclear protein, was negatively correlated to short-term efficacy and long-term survival of patients with ESCC receiving radical RT (**Tables 1**, **2**). Meanwhile, a positive correlation trend between NRAGE and β-catenin nuclear expression were also observed using the Spearman analysis (**Table S3**). Comprehensively, the current results confirmed that IR may cause the upregulation of NRAGE, which could accumulate NRAGE to promote nuclear translocation, and triggered β-catenin nuclear accumulation to induce proliferation and anti-proptosis of the ESCC cells, enhance invasiveness and migration capability and cell cycle rearrangement, and promote decreased radiosensitivity (**Figure 6S**).

## Conclusion

Collectively, our study verified the NRAGE, with anti-oncogene and oncogene contradictory roles, was regarded as an oncogene due to the functions that accelerated proliferation, anti-apoptosis effect, more malignant migration and invasion, and accumulation of IR resistance by triggering Wnt/β-catenin signaling pathway in ESCC cells in 2D and 3D levels. Not only that, clinical correlation analysis also demonstrated that NRAGE, specifically for NRAGE nuclear protein, was a risk factor in patients with ESCC treated by definitive RT and had a positive relationship with β-catenin nuclear protein expression. As a putative oncogene, NRAGE may have the potential to serve as a novel predictive biomarker for tumor progression and target of molecular therapy.

## Data Availability Statement

The original contributions presented in the study are included in the article/**Supplementary Material**. Further inquiries can be directed to the corresponding author.

## Ethics Statement

The studies involving human participants were reviewed and approved by the Ethics Committee of the Second Hospital of Hebei Medical University. The patients/participants provided their written informed consent to participate in this study. Written informed consent was obtained from the individual(s) for the publication of any potentially identifiable images or data included in this article.

## Author Contributions

HZ and GW is attributed for the study design, data analysis, and manuscript writing. ZX established cell lines, tissue collection, and evaluation of radiotherapy efficacy. ZX, YY, and ZT helped interpreting the data. CG, XH, WS, and LH prepared all the figures. JL, HZ, and GW edited all the tables. XX is attributed for the experiment conduction. All authors listed have made a substantial, direct, and intellectual contribution to the work and approved it for publication.

## Funding

This work is supported by a grant from the Natural Science Foundation of Hebei Province (No. H2019206182).

## Conflict of Interest

The authors declare that the research was conducted in the absence of any commercial or financial relationships that could be construed as a potential conflict of interest.

## Publisher’s Note

All claims expressed in this article are solely those of the authors and do not necessarily represent those of their affiliated organizations, or those of the publisher, the editors and the reviewers. Any product that may be evaluated in this article, or claim that may be made by its manufacturer, is not guaranteed or endorsed by the publisher.
