# Supplementary material for: NRAGE Confers Radiation Resistance in 2D and 3D Cell Culture and Poor Outcome in Patients With Esophageal Squamous Cell Carcinoma
Source: Front Oncol. 2022 Apr 1;12:831506. doi: 10.3389/fonc.2022.831506 (PMC9010827; doi:10.3389/fonc.2022.831506)
Supplement: Supplementary file 1 [file DataSheet_1.docx]

Supplementary Material

# Supplementary methods

## Cell culture

The human ESCC cell line Eca109 was purchased from the Institute of iCell Bioscience Inc (Shanghai, China), and were cultured at 37°C and 5% CO2 with RPMI-1640 medium (Gibco, Life Technologies) appending 10% fetal bovine serum (AusGeneX, Australia), 100 U/mL penicillin, 100 mg/mL streptomycin. Cells were passaged when reaching 70% to 80% confluence.

## Plasmids and stable transfection

The genes encoding human NRAGE (NM_001005333)-overexpression recombinant plasmid construction and DNA preparation followed standard procedures for In-Fusion™ Advantage PCR Cloning Kit (Clontech, Japan). Primers for NRAGE cDNA: upstream, 5′-ATGGCTCAGAAAATGGACTGTGGT-3′; down-stream, 5′-CTCAACCCAGAAGAAACCAATGGC-3′; An empty-vector construct was used as the control. Briefly, first day, Eca109 cells were seeded into a 6-well plate with 1×10^5^ cells/well, and then NRAGE recombinant plasmid and empty-vector were transfected into cells using lipofectamine^TM^ 2000 (Invitrogen, USA) reagent. After 24h, Transfected cells were screened against 500μg/mL G418 (Solarbio, Beijing, China) for 14 days. Monoclonal Eca109-overexpression NRAGE (E/N) and Eca109-overexpression empty-vector (E) cells were obtained by limited dilution screening.

## Realtime PCR

Total RNA was extracted from the cells with Trizol reagent (Invitrogen, USA) according to the manufacturer’s instructions. Reverse transcribed reaction was performed using RevertAid^TM^ First Strand cDNA Synthesis Kit (Thermo Scientific, K1622, USA). Expression of NRAGE mRNA was performed with realtime PCR using SYBR Premix ExTaq™ (Takara, Shiga, Japan) on CFX 96™ Real-Time PCR Detection System (BIO-RAD Laboratories, Inc., California, USA). GAPDH was used as the internal control. The [design](javascript:;) [formulas](javascript:;) of fold changes was 2 ^− [(Ct of target gene) − (Ct of GAPDH)]^ method. The primers were as followed, NRAGE

Forward:5’- GCTCGGTCTCCTCTTGGTGATTC-3’;

Reverse: 5’-GGCACTCGTCTGTAGTCCAGGTATT-3’.

## Western blot

Cells were collected and then lysed for 20 mins in cold lysis buffer (Beyotime Institute of Biotechnology, China) containing Protein phosphatase inhibitors (protein Phosphatase Inhibitor, All-in-one, 100 ×, Solarbio, Beijing, China). BCA kit (Generay, Shanghai, China) was used to detected protein concentrations; 20μg of samples were resolved by 10% SDS-PAGE and then transferred to polyvinylidene fluoride (PVDF) as previously described. Protein bands were visualized using enhanced chemiluminescent substrate according to the manufacturer’s protocol (Millipore, Billerica, MA, USA). The primary antibodies against NRAGE (Proteintech, Wuhan, China), β-catenin (Proteintech, Wuhan, China), p-β-catenin (CST, USA), Gsk3β (Proteintech, Wuhan, China), p-Gsk3β (CST, USA), GAPDH (ZSGB-BIO, Beijing, China), Cyclin D1 (Affinity Biosciences. OH. USA) were used.

## CCK-8 assay

E and E/N on proliferation was evaluated by cell counting kit-8 (CCK-8, MedChemExpress, USA) assay. Briefly, after irradiated with 2 Gy, 5 Gy or not, 1×10^5^ cells/well of E and E/N cells were seeded into 96-well plates in 100 mL R1640 medium and incubated at 37℃ with 5% CO2. Culture the cells in a CO_2_ incubator at 37°C for 24 hours. 10 μL of CCK-8 solution was added in each corresponding well using a repeating pipettor. Incubate the plate for 1~4 hours in the incubator. After that, the absorbances at 450 nm were measured by Multi-function microporous plate detector (Biotek Synergy-H1, USA).

## Irradiation and Clonogenic Assay

The radioresistance was measured via colony formation assays following exposure to IR. After being seeded in 6-well plates at 100, 100, 300, 500 cells/well and in 10-mm culture dishes at 3000 or 5000 cells/dish, E and E/N Cells were treated with various doses of radiation (0, 2, 4, 6, 8 or 10 Gy) of 6MV X-rays by a linear accelerator (Elekta, Synergy) at a dose rate of 200 cGy/min using a tissue compensation system with a 1.5-cm membrane. Then, the cells were incubated for 10~14 days until colonies appeared. After being fixed with methanol for 20 min and stained with 0.1% crystal violet (Solarbio, Beijing, China) for15 min. a valid clone was containing at least 50 cells. Experiments were performed in triplicate. plating efficiency (PE)= number of valid clones /numbers of corresponding cells inoculated; Surviving fraction (SF)=PE experiment group/PE sham-irradiated group; GraphPad Prism 8.0 software was used to fit the data to a multi-target, single-hit model (y = 1-(1-exp(-k*x)) ˆN).

## Wound Healing assay

After irradiated by 5Gy or not, E and E/N were seeded into 6-well plate (2×10^5^ cells/Wells), in triplicate, for the wound healing assay. After 24 h of culture (T0), the "1" glyph was scratched on the bottom of the culture plate, followed by washing gently three times with PBS and addition of culture medium with 2.5% FBS. The scratches were immediately observed under the microscope and photographed. After continuing culture for 12 and 24 hours, additional photographs were taken. Image J software was used to analyze data and calculate cell mobility. Mobility = (scraped area at T0 - scraped area at each time point)/ scraped area at T0 ×100%.

## Transwell Invasion Assay

100μL cell suspension without FBS containing 2×10^4^ cells at logarithmic phase were collected and added to the upper compartment of Transwell chamber (8-μm pore size; Corning, USA). 600 μL medium containing 10% FBS was added to 24-well plate and cultured for 24 hours. The cells on the top surface and matrigel were wiped off with cotton swabs. After fixation by 4% paraformaldehyde for 15 min, stained with 0.1% crystal violet for 15 min, washed with PBS, photographed under microscope (100×), and count the number of cells through the membrane.

## Flow cytometry analysis

E and E/N cells with or without IR were suspended in culture medium at a concentration of 5×10^6^ cells mL^-1^, For apoptosis, 5µL Annexin-V and PI staining solution (Solarbio, Beijing, China) were added to 500 µL of cell suspension for 15 min incubation at room temperature without light, stained cells were assayed and quantified using a FACS Flow Cytometer (FCM, BD, USA); For cell cycle, after preparing the cells by the same method, 500μL ice-cold 70% ethanol was added to fix cells at 4°C for the night. Washed cells by ice-cold PBS, added 100μL RNase A solution to resuspend the cells, and incubated at 37℃ for 30min. Followed by the addition of 400µl PI for 20 min at 4℃ and cell cycle was analyzed by FCM.

## γ-H2AX Immunofluorescence Staining

γ-H2AX foci immunofluorescence staining was used to analyze the DNA double-strand breaks (DSBs) kinetics. Cells were treated with 5 Gy IR at logarithmic growth phase. Then, cells were fixed with 4% paraformaldehyde for 30 minutes at 0.5h, 2h, 6h after washing with PBS. After permeabilized with 0.2% Triton X-100 for 20 minutes, cells were stained for γ-H2AX foci (1:100, 2577S, CST, USA) for 2h at room temperature. And secondary antibody (1:100, Abcam, USA) for 1 h at room temperature in the dark. DAPI (Solarbio, Beijing, China) was to stained the nuclei for 20 min at room temperature. Fluorescence microscope (Leica Microsystems) acquire images. Finally, Image J software was used to count the γ-H2AX fluorescent spots in nucleus.

## Histology and immunohistochemistry

ESCC specimens from tissues derived from patients or 3D printed scaffolds of E and E/N cells were fixed in 4% paraformaldehyde for 24h. These tissues and scaffolds were embedded in paraffin. And then, part of the scaffolds was cut into 2μm thick sections, and stained with haematoxylin and eosin (HE) following the protocol. In addition, these tissues and scaffolds were cut into 5-μm thick sections. According to the protocol, the universal streptavidin-peroxidase kit (ZSGB-bio, Beijing, China) was used to detect the protein expression of NRAGE, β-catenin and Ki-67(NRAGE, 1:100, Proteintech, China; β-catenin, 1:150, Proteintech, China; Ki-67, ZSGB-BIO, China). Brown granules in the cytoplasm, cell membrane, or nucleus were considered as positive staining. According to the Axiotis pathological scoring standard, the percentage of positive cells and the staining intensity of cells were comprehensively evaluated. Two pathologists observed independently 5 representative high-magnification fields of each section, and scored them according to the staining intensity and percentage of staining cells in the tumor cytoplasm or nucleus: 0 for negative, 1 for light staining, 2 for moderate staining, and 3 for deep staining. The percentage of staining cells in counting cells <5% was 0 points, 6%-25% was 1 point, 26%-50% was 2 points, 51%-75% was 3 points, and 76%-100% was 4 points. The final score of each slice was obtained by multiplying the two score. 0 was negative (-), 1~4 was weak positive (+), 5~8 was positive (++), and 9~12 was strongly positive (+++).

# Supplementary Tables

## Supplementary Tables1

| Table S1 Correlation parameters in the multi-target, single-hit model | | | |
| --- | --- | --- | --- |
| Group | SF2 | D0 | Dq |
| E | 0.518 | 1.201 | 1.492 |
| E/N | 0.636 | 2.020 | 1.530 |

D0 is the single radiation dose of radiation producing a 63% lethality rate; SF2 is the survival fraction with 2 Gy radiation;

Dq is the quasi threshold dose required for sublethal damage.

## Supplementary Tables2

Table S2 clinicopathological characteristics of patient samples with ESCC following Radical radiotherapy

| Characteristics | Number of cases (%) |
| --- | --- |
| Age(y) |  |
| ≥60 | 29（65.9） |
| <60 | 15（34.1） |
| Gender | |
| Male | 29（65.9） |
| Female | 15（34.1） |
| Clinical Stage | |
| I-II | 33（75） |
| III-IV | 11（25） |
| Tumor size | |
| ≥5cm | 30（68.2） |
| ＜5cm | 14（31.8） |
| lymph nodes metastasis (LNM) | |
| Yes | 26（59.1） |
| No | 18（40.9） |
| Synchronous chemotherapy | |
| Yes | 29（65.9） |
| No | 15（34.1） |
| Curative efficacy | |
| effectivity | 36（81.8） |
| inefficacy | 8（18.2） |
| Events | |
| [Censored](javascript:;) | 18(40.9) |
| Dead | 26(59.1) |
| Expression of NRAGE | |
| Weak positive | 17(38.64) |
| Positive | 18(40.91) |
| Strongly positive | 9(20.45) |
| Expression of NRAGE nuclear | |
| Weak positive | 21(47.73) |
| Positive | 17(38.64) |
| Strongly positive | 6(13.64) |
| Expression of β-catenin | |
| Weak positive | 23(52.27) |
| Positive | 16(36.36) |
| Strongly positive | 5(11.36) |
| Expression of β-catenin nuclear | |
| Weak positive | 33(75) |
| Positive | 7(15.91) |
| Strongly positive | 4(9.09) |

## Supplementary Tables3

Table S3 The linear correlation between NRAGE nuclear protein and β-catenin nuclear protein

|  |  | **Expression of β-catenin nuclear** | | | **Spearman**  **correlation** | ***P*** |
| --- | --- | --- | --- | --- | --- | --- |
|  |  | **Weak positive** | **Positive** | **Strongly positive** |  |  |
| **Expression of NRAGE nuclear** | **Weak positive** | 18 | 3 | 0 | 0.291 | 0.055 |
|  | **Positive** | 12 | 2 | 3 |  |  |
|  | **Strongly positive** | 3 | 2 | 1 |  |  |
